# Achiasmatic meiosis in the unisexual Amazon molly, *Poecilia formosa*

**DOI:** 10.1007/s10577-022-09708-2

**Published:** 2022-12-02

**Authors:** Dmitrij Dedukh, Irene da Cruz, Susanne Kneitz, Anatolie Marta, Jenny Ormanns, Tomáš Tichopád, Yuan Lu, Manfred Alsheimer, Karel Janko, Manfred Schartl

**Affiliations:** 1 Laboratory of Non-Mendelian Evolution, Institute of Animal Physiology and Genetics, Czech Academy of Sciences, Rumburská 89, Liběchov, 277 21 Czech Republic; 2 Developmental Biochemistry, Biocenter, University of Wuerzburg, Am Hubland, 97074 Wuerzburg, Germany; 3 Biochemistry and Cell Biology, Biocenter, University of Wuerzburg, Am Hubland, 97074 Wuerzburg, Germany; 4 Institute of Zoology, Academiei 1, 2001, MD-2028 Chisinau, Moldova; 5 Faculty of Fisheries and Protection of Waters, South Bohemian Research Center of Aquaculture and Biodiversity of Hydrocenoses, University of South Bohemia in České Budějovice, Zátiší 728/II, 389 25 Vodňany, Czech Republic; 6 Xiphophorus Genetic Stock Center, Texas State University, San Marcos, TX 78666 USA; 7 Cell and Developmental Biology, University of Wuerzburg, Am Hubland, 97074 BiocenterWuerzburg, Germany; 8 Department of Biology and Ecology, Faculty of Science, University of Ostrava, Chittussiho 10, 710 00 Ostrava, Czech Republic

**Keywords:** Meiosis, Parthenogenesis, Synaptonemal complex, Recombination, Crossing-over, Achiasmatic, Transcriptome, Oogenesis

## Abstract

Unisexual reproduction, which generates clonal offspring, is an alternative strategy to sexual breeding and occurs even in vertebrates. A wide range of non-sexual reproductive modes have been described, and one of the least understood questions is how such pathways emerged and how they mechanistically proceed. The Amazon molly, *Poecilia formosa*, needs sperm from males of related species to trigger the parthenogenetic development of diploid eggs. However, the mechanism, of how the unreduced female gametes are produced, remains unclear. Cytological analyses revealed that the chromosomes of primary oocytes initiate pachytene but do not proceed to bivalent formation and meiotic crossovers. Comparing ovary transcriptomes of *P. formosa* and its sexual parental species revealed expression levels of meiosis-specific genes deviating from *P. mexicana* but not from *P. latipinna*. Furthermore, several meiosis genes show biased expression towards one of the two alleles from the parental genomes. We infer from our data that in the Amazon molly diploid oocytes are generated by apomixis due to a failure in the synapsis of homologous chromosomes. The fact that this failure is not reflected in the differential expression of known meiosis genes suggests the underlying molecular mechanism may be dysregulation on the protein level or misexpression of a so far unknown meiosis gene, and/or hybrid dysgenesis because of compromised interaction of proteins from diverged genomes.

**Supplementary Information:**

The online version contains supplementary material available at 10.1007/s10577-022-09708-2.

## Introduction

Asexuality is an exceptional and rare mode of reproduction in vertebrates. Understanding the genetic basis and molecular mechanisms of asexuality and why it persists in some species is not only of interest to comprehend the biology of those peculiar biotypes, but also contributes to the understanding of the mechanisms and evolution of sexual reproduction. Asexuality has the advantage of a faster exponential population growth (Loewe and Lamatsch 2008) and the avoidance of the two-fold cost of sex (Maynard Smith 1978). However, generating genetically identical individuals has main disadvantages: its consequences are low genetic diversity (known as the “Red Queen” effect) and a decay of fitness due to accumulation of deleterious mutations (described by “Muller’s ratchet”) (Van Valen 1973; Bell 1982). These drawbacks are considered to outweigh the positive features of clonal propagation (Lynch et al. 1995; Neiman et al. 2010; Lively and Morran 2014). In addition to the predicted importance of those negative consequences, considerations that the changes in cellular mechanisms required for asexual reproduction are complicated are generally taken as explanation for the rarity of parthenogenetic vertebrates. It was only in 1932, when the Amazon molly, *Poecilia formosa*, was detected as the first vertebrate to reproduce by “a form of apparent parthenogenesis” in nature (Hubbs and Hubbs 1932). To date, approximately 100 fish, amphibian and reptile biotypes are known to reproduce obligatory or transiently by parthenogenesis, while the phenomenon, as a natural reproductive process, appears to be absent in endotherm vertebrates (Avise 2008; Stöck et al. 2021).

The so-called asexual organisms in general, and vertebrates in particular, employ a plethora of varying cytological mechanisms to achieve production of unreduced gametes or at least partially clonal inheritance. Amongst these, several mechanisms appear to have emerged many times independently amongst unrelated lineages (Stenberg and Saura 2009). One such mechanism assumes suppression of karyokinesis and cytokinesis in oogonia during the last premeiotic mitosis. This yields a primary oocyte with twice the number of chromosome sets, which form twice the number of bivalents during pachytene, followed by two regular meiotic divisions. This process, termed pre-meiotic endoreplication, results in an ovum with the somatic number of chromosomes and clonal genomic constitution. Because of the occasional pairing of homologous chromosomes, which then recombine, it is an automictic form of asexuality. It is relatively widespread amongst clonal animals (Stenberg and Saura 2009). In vertebrates, it occurs in triploid forms of some *Ambystoma* salamanders (Macgregor and Uzzell 1964; Cuellar 1976), diploid and triploid lizards from the genus *Aspidoscelis* (Cuellar 1971), *Lepidodactylus*, *Hemiphyllodactylus* and *Heteronotia* (Dedukh et al. 2022), in Batura toads (Stöck et al. 2002), and in teleost fishes in triploid *Poeciliopsis monacha 2-lucida* (Cimino 1972), in diploid and triploid hybrids of *Cobitis* (Dedukh et al. 2020) and *Misgurnus* species (Itono et al. 2006). Another automictic mechanism that can restore a diploid egg while retaining meiosis during the parthenogenetic development is fusion of the oocyte after the first reduction division with the first or second polar body. This mechanism explains the process of facultative parthenogenesis observed in some sharks (Chapman et al. 2007), Komodo dragons and snakes (Card et al. 2021). It is widespread amongst plants and is known from insects, crustaceans and tardigrades (Stenberg and Saura 2009). Interestingly, this automictic process has been observed regularly in laboratory-produced hybrids of *P. mexicana* and *P. latipinna* (Lampert et al. 2007) and could be seen as the primordial mechanism from which oogenesis in *P. formosa* evolved. Finally, asexuality can be fully apomictic with meiosis being totally suppressed and the oocyte being produced by mitosis. Apomixis does not lead to genetic variation in the resultant clonal population. Automixis by fusion of the products of the meiotic division, however, can lead to variable offspring.

Since its discovery *P. formosa* became a paradigmatic model for studies on asexuality (Schlupp 2005; Lampert and Schartl 2008). It is an all-female species, which reproduces by gynogenesis, a form of parthenogenesis. This mode of reproduction still requires the presence of males. Males of related sexual species provide sperm as a trigger to physiologically activate the development of the embryo from a diploid egg without contributing genetically to the offspring. The sperm DNA is usually degraded before karyogamy can occur, which guarantees clonality—with only extremely rare exceptions. Minute parts of the paternal genome can persist as microchromosomes, which behave like B-chromosomes in the soma and the germline. In other rare cases, the sperm exclusion mechanism fails completely, and a triploid Amazon molly develops (for reviews on the reproductive biology of *P. formosa* see Schlupp 2005; Lampert and Schartl 2008). Genealogically, *P. formosa* is derived from two sexual species. All individuals of today’s *P. formosa* stem from a single interspecific hybrid of two distantly related *Poecilia* species, *P. mexicana* as the maternal and *P. latipinna* as the paternal ancestor (Stöck et al. 2010; Warren et al. 2018)*.*

However, despite *P. formosa* being a paradigmatic organism for studies on the origins and evolutionary consequences of asexuality, there is no consensus how sexual reproduction was lost in the “prima Eva” of all Amazon mollies and how diploid clonal lineages are perpetuated. For this, several hypotheses can be envisaged in analogy to the aforementioned cases from other asexual animals. With respect to the mechanism of oocyte formation in *P. formosa*, cytological data were first interpreted as evidence for premeiotic endoreduplication (Hubbs and Hubbs 1932; Rasch et al. 1982). Later, however, on basis of cytophotometry data and the absence of synapsed chromosomes in cytological and electron microscopic preparation this hypothesis was rejected and apomixis was ascribed to the Amazon molly (Monaco et al. 1981, 1984; Rasch et al. 1982). A transcriptomic study found a general downregulation of genes related to meiosis and reproduction in the Amazon molly ovary (Schedina et al. 2018). Despite, the cytogenetic and molecular information to infer the mechanism how unreduced oocytes are produced is lacking. In this work, we show by cytogenetic analyses using immunodetection of synaptonemal complex components, sites of double-strand breaks (DSB) repair and meiotic crossovers markers that the chromosomes of *P. formosa* primary oocytes take the preparatory steps of meiosis I but do not proceed to bivalent formation and homologous recombination. Furthermore, transcriptome analyses uncovered that most genes are expressed in a similar pattern in the asexual ovary of *P. formosa* and ovaries of both sexual parental species. The expression profiles of genes with a known function in meiosis are almost perfectly reflecting those of *P. latipinna*, while some key genes from prophase I are differentially expressed in *P. formosa* in comparison to the sexual species *P. mexicana*.

## Materials and methods

### Animals

All fish were reared under standard conditions for poeciliid fish husbandry (Kallman 1975) with a light/dark cycle of 14/10 h at 26 °C in the fish facility of the Biocentre at the University of Würzburg, Germany. Animals were kept and sampled in accordance with the applicable EU and national German legislation governing animal experimentation. In particular, all experimental protocols were approved through an authorization (FB VVL 568/201–2792/20) of the Veterinary Office of the District Government of Lower Franconia, Germany, in accordance with the German Animal Protection Law (TierSchG).

Fish from the following laboratory lines were used:

- *Poecilia formosa*, WLC #4698 (laboratory strain PfI), #4394 (origin Rio Purificacion, Tamps. Mexico VI/17), #1341 (origin Ciudad Mante, Tamps. Mexico III/2), #1304 (laboratory strain PfII)
- *Poecilia mexicana*, WLC#1353 (origin Laguna del Champayan, Tamps. Mexico IV/5)
- *Poecilia latipinna*, WLC#1442 (origin Tampico, Tamps, Mexico IX/24)

Hybrids: Ovary transcriptome data from a previous study analysing F1 hybrids from crossing virgin *P. mexicana* female with a *P. latipinna* male (Lu et al. 2021) were used.

### RNA extractions and transcriptome sequencing

Ovaries from four adult non-pregnant *P. formosa*, *P. latipinna* and *P. mexicana* were dissected at day 0–3 after giving birth during the time when the next clutch of oocytes matures. Total RNA was isolated using TRIzol reagent (Thermo Fisher Scientific, Waltham, USA) according to the supplier’s recommendation. Custom eukaryotic strand-specific sequencing (BGI, Shenzen, China) of TruSeq libraries generated 30–35 million 150 bp paired end clean reads for each sample on the BGISEQ platform. Besides the four biological replicates, one technical replicate of *P. formosa* and *P. latipinna* was sequenced. To validate the replicates, a PCA analysis was done by DESeq2 (Love et al. 2014).

### Differential gene expression analysis

After duplicate and barcode removal reads were aligned to the *P. formosa* genome version 5.1.2 (GCA_000485575.1) using the STAR aligner version 2.5 (–runMode alignReads –quantMode GeneCounts) (Dobin et al. 2013). A gene was considered as expressed if it had more than 10 reads aligned in each sample. Based on the counts of the aligned reads, differentially expressed genes between *P. formosa*, *P. mexicana*, *P. latipinna* and F1 hybrids (*Pmex/P.lat*) were obtained using DESeq2 (Love et al. 2014). A gene was defined as differentially expressed if the log2 fold change (FC) is >|2| and a *p*-adj < 0.05. For focussing on genes with known function during meiosis and germ cell development gene, we retrieved the IDs of the female meiotic genes from the meiosis online database (https://mcg.ustc.edu.cn/bsc/meiosis/index.html) (Li et al. 2014). Functional clustering was performed by the Database for Annotation, Visualization and Integrated Discovery (DAVID, https://david.ncifcrf.gov/) using human homologues.

### Allele biased expressed gene analysis

To evaluate the differences in relative expression between parental alleles, the reads from *P. formosa* ovaries were mapped using Bowtie2 (Langmead and Salzberg 2012) against both parental transcriptomes (i.e. cds of *P. latipinna* and *P. mexicana*) that are orthologous to *P. formosa* (Lu et al. 2021). Uniquely mapped reads were retrieved by filtering the low mapping quality (< 15) using SAMtools (Li et al. 2009) and transcript abundance was estimated as transcripts per million (TPM) using Salomon (Patro et al. 2017). Genes with TPM ≥ 1 in all samples were considered as expressed and were included in further analyses.

The frequency of parental allele biased expression for all expressed genes and for meiotic genes was estimated as follows:

$$A_{Plat}=\sum{TPM}_{Plat}/\left(\sum{TPM}_{Plat}+\sum{TPM}_{Pmex}\right)$$

$$A_{Plat}=\sum{TPM}_{Pmex}/\left(\sum{TPM}_{Pmex}+\sum{TPM}_{Plat}\right)$$

where *A* is *P. latipinna* or *P. mexicana* parental allele and *TPM* is the expression value determined for each parental allele. A gene was determined as *P. latipinna* or *P. mexicana* allele expression biased if the frequency was over 60%.

Protein interaction network was produced for the meiosis genes identified with allelic expression bias by applying the STRING database and visualized using Cytoscape software 3.9.0 (Shannon et al. 2003; von Mering et al. 2003).

### Pachytene chromosomes and immunofluorescent staining

Pachytene chromosomes were obtained from juvenile females (1–14 days after birth) using a modification of the protocol described in Peters et al. (1997). Ovaries were placed in 100 µl of 100 mM sucrose and incubated for 10 min followed by preparation of cell suspension. The suspension was immediately dropped on SuperFrost® slides (Menzel Gläser) which had been dipped in a fresh 1% paraformaldehyde (PFA) pH 8.5 solution containing 0.15% Triton X-100. Cells were distributed by gentle inclination of the slide. Slides were placed in a humid chamber for at least 1 h. Afterward, slides were dried, washed in phosphate buffered saline (1 × PBS; 4.3 mM Na_2_HPO_4_, 1.43 mM KH_2_PO_4_, 2.7 mM KCl, 137 mM NaCl, pH 7.4) and stored at 4 °C in 1 × PBS for immunofluorescent staining procedure not longer than 1 week.

The following primary antibodies were used to detect lateral and central components of synaptonemal complexes (SC): rabbit polyclonal antibodies (ab14206, Abcam) against Sycp3 protein and chicken polyclonal antibodies (a gift from Sean M. Burgess (Blokhina et al. 2019)) against Sycp1 protein correspondingly. Sites of DSB repair were visualized by chicken polyclonal antibodies against Rad51 recombinase (GeneTex GTX00721). Cross-over sites were detected with mouse monoclonal antibodies (ab 15,095, Abcam) against Mlh1 protein. Prior to adding the antibodies, fresh slides were incubated with 1% blocking reagent (Roche) in 1 × PBS containing 0.01% Tween-20 for 20 min. Antibodies were added in concentrations according to the manufacturers’ specifications for 1 h at RT. Slides were washed three times in 1 × PBS at RT and incubated with secondary antibodies Alexa-488-conjugated goat anti-rabbit IgG (H + L) (Thermo Fisher Scientific), Alexa-594 goat anti-chicken IgY (H + L) (Thermo Fisher Scientific) and Alexa-594-conjugated goat anti-mouse IgG (H + L) (Thermo Fisher Scientific) for 1 h at RT. Slides were washed three times in 1 × PBS and mounted in Vectashield/DAPI (1.5 mg/ml) (Vector, Burlingame, CA, USA).

### Diplotene chromosome isolation

Diplotene chromosomal spreads (also known as “lampbrush chromosomes”) were prepared from *P. mexicana* and *P. formosa* females according to an earlier published protocol initially developed for amphibians (Gall et al. 1991) with slight modifications. Ovaries from non-stimulated females were dissected and placed in the OR2 saline medium (82.5 mM NaCl, 2.5 mM KCl, 1 mM MgCl_2_, 1 mM CaCl_2_, 1 mM Na_2_HPO_4_, 5 mM HEPES (4-(2-hydroxyethyl)-1-piperazineethanesulfonic acid); pH 7.4). Individual oocytes were transferred to the isolation medium “5:1” (83 mM KCl, 17 mM NaCl, 6.5 mM Na_2_HPO_4_, 3.5 mM KH_2_PO_4_, 1 mM MgCl_2_, 1 mM DTT (dithiothreitol); pH 7.0–7.2) where oocyte nuclei were isolated manually using jeweller forceps (Dumont). Oocyte nuclei were washed in one-fourth strength “5:1” medium with the addition of 0.1% paraformaldehyde and 0.01% 1 M MgCl_2_ and transferred to glass chambers attached to a slide filled in one-tenth strength “5:1” medium. Afterward, the nuclear membrane was carefully disrupted allowing the release of nucleoplasm into the medium. Thus, each chamber contained chromosomal spread from the individual oocyte. The slide was subsequently centrifuged for 20 min at + 4 °C, 4000 rpm, in a Hettich Universal 320 centrifuge equipped with 2-Place swing bucket rotor for plates, fixed for 30 min in 2% paraformaldehyde in 1 × PBS, and post-fixed in 70% ethanol overnight (at + 4 °C).

### Confocal laser scanning microscopy

In addition to the classical analysis of diplotene chromosomal spread, we investigated the intact oocyte nucleus from *P. formosa* and *P. mexicana* individuals. Nuclei were isolated with fine forceps from oocytes of 0.5–1 mm diameter in isolation medium “5:1”. Isolated nuclei were transferred to isolation medium containing 1 µM TO-PRO™-3 Iodide (Thermo Fisher Scientific). Confocal laser scanning microscopy was conducted with a Leica TCS SP5 microscope based on a Leica DMI 6000 CS inverted microscope. Specimens were examined by the XYZ scanning technique using HC PL APO 20 × objective and HeNe laser (633 nm).

Images were captured and processed using LAS AF software (Leica Microsystems, Germany); 3D reconstructions were made using Imaris 5.0.1 (Bitplane, AG) software.

### Wide-field and fluorescence microscopy

Immunostained meiotic chromosomes were analysed using a Provis AX70 Olympus microscope equipped with standard fluorescence filter sets. Microphotographs of chromosomes were captured by a CCD camera (DP30W Olympus) using Olympus Acquisition Software. Microphotographs were finally adjusted and arranged in Adobe Photoshop, CS6 software.

## Results

### Bivalent formation does not occur during meiosis of the asexual Poecilia formosa

**Fig. 1:**
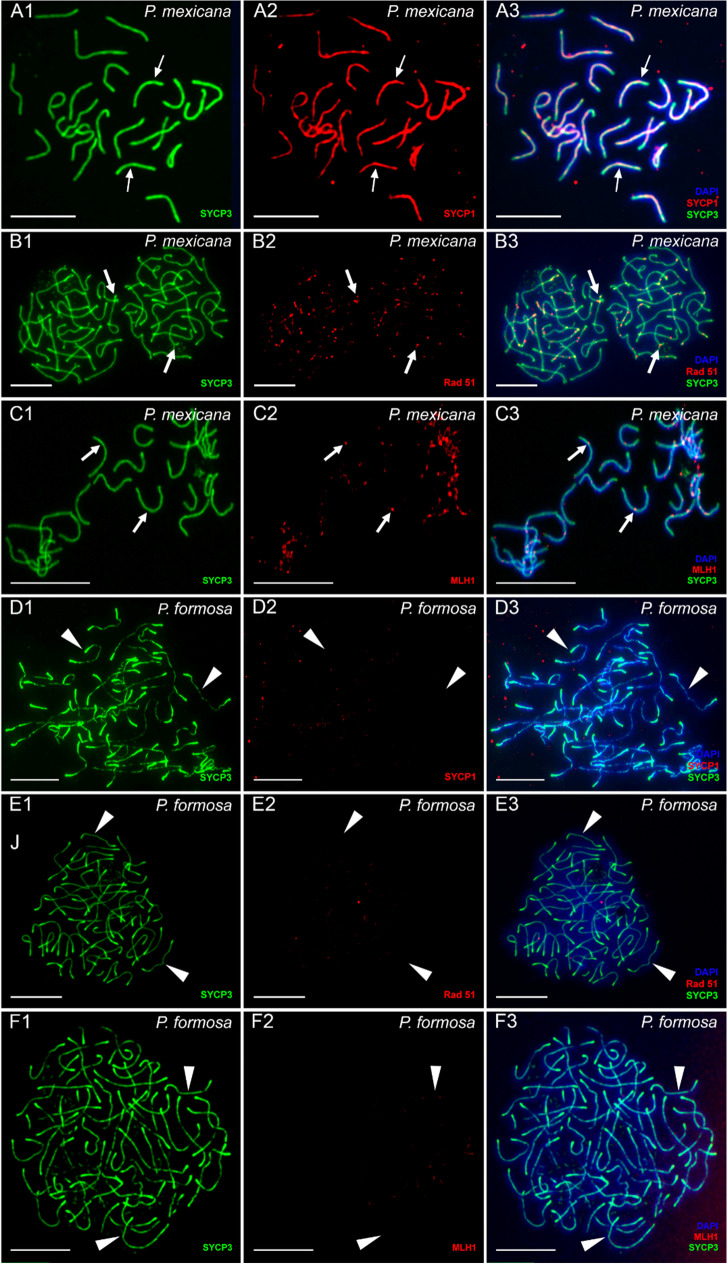
Pachytene chromosome spreads of *P. mexicana* (**A1**–**C3**) and *P. formosa* (**D1**–**F3**). Staining for lateral (SYCP3) and central (SYCP1) components of synaptonemal complexes clearly show the presence of 23 bivalents (indicated by arrows) in *P. mexicana* (**A1**, **A2**, **A3**) and 46 univalents (indicated by arrowheads) in *P. formosa* (**D1**, **D2**, **D3**). RAD 51 immunostaining (thick arrows) shows the presence of DSBs formation in *P. mexicana* bivalents (**B1**, **B2**, **B3**) but not in *P. formosa* univalents (**E1**, **E2**, **E3**). MLH1 immunostaining (thick arrows) demonstrates the occurrence of crossovers in *P. mexicana* bivalents (**C1**, **C2**, **C3**) while no crossovers were detected in *P. formosa* univalents (**F1**, **F2**, **F3**). Scale bar = 10 µm

To investigate the ability of *P. formosa* females to form bivalents, we performed immunostaining of chromosomal spreads during pachytene with antibodies against central (Sycp1) and lateral (Sycp3) components of the synaptonemal complex (SC). It has been shown that Sycp3 is localized on both bivalents and univalents while Sycp1 accumulates only on bivalents (Iwai et al. 2006; Bisig et al. 2012; Blokhina et al. 2019). In the sexual *P. mexicana* females, representing the maternal ancestor of *P. formosa*, we detected normal pairing and formation of 23 bivalents with no evidence of univalents or aberrant pairing (Fig. 1A1–A3). To check for the presence of homologous recombinational repair of DSBs, antibodies against Rad51 proteins (Neale and Keeney 2006; Smagulova et al. 2011) were used (Fig. 1B1–B3). All bivalents of *P. mexicana* showed one to two Rad51 foci (Fig. 1B1–B3). In addition, we used antibodies against Mlh1 proteins to detect meiotic crossover sites on synapsed chromosomes (Kolas et al. 2005; Moens 2006). One to two crossover spots per bivalent were observed (Fig. 1C1–C3). Conversely, in *P. formosa* we detected accumulation only of Sycp3 but not Sycp1 protein, suggesting that the process of synapsis is incomplete and, consequently, bivalents are not formed during pachytene stage (Fig. 1D1–D3). We observed 46 univalents in accordance with the diploid number of chromosomes in this species (Fig. 1D1–D3). Consistent with this, on pachytene spreads from *P. formosa* oocytes stained with an antibody against Rad51 no foci were detected (Fig. 1E1–E3) suggesting that DSBs repair is not occurring.

Moreover, on univalents of *P. formosa*, we did not observe Mlh1 foci which further supports the absence of crossovers (Fig. 1F1–F3).

**Fig. 2:**
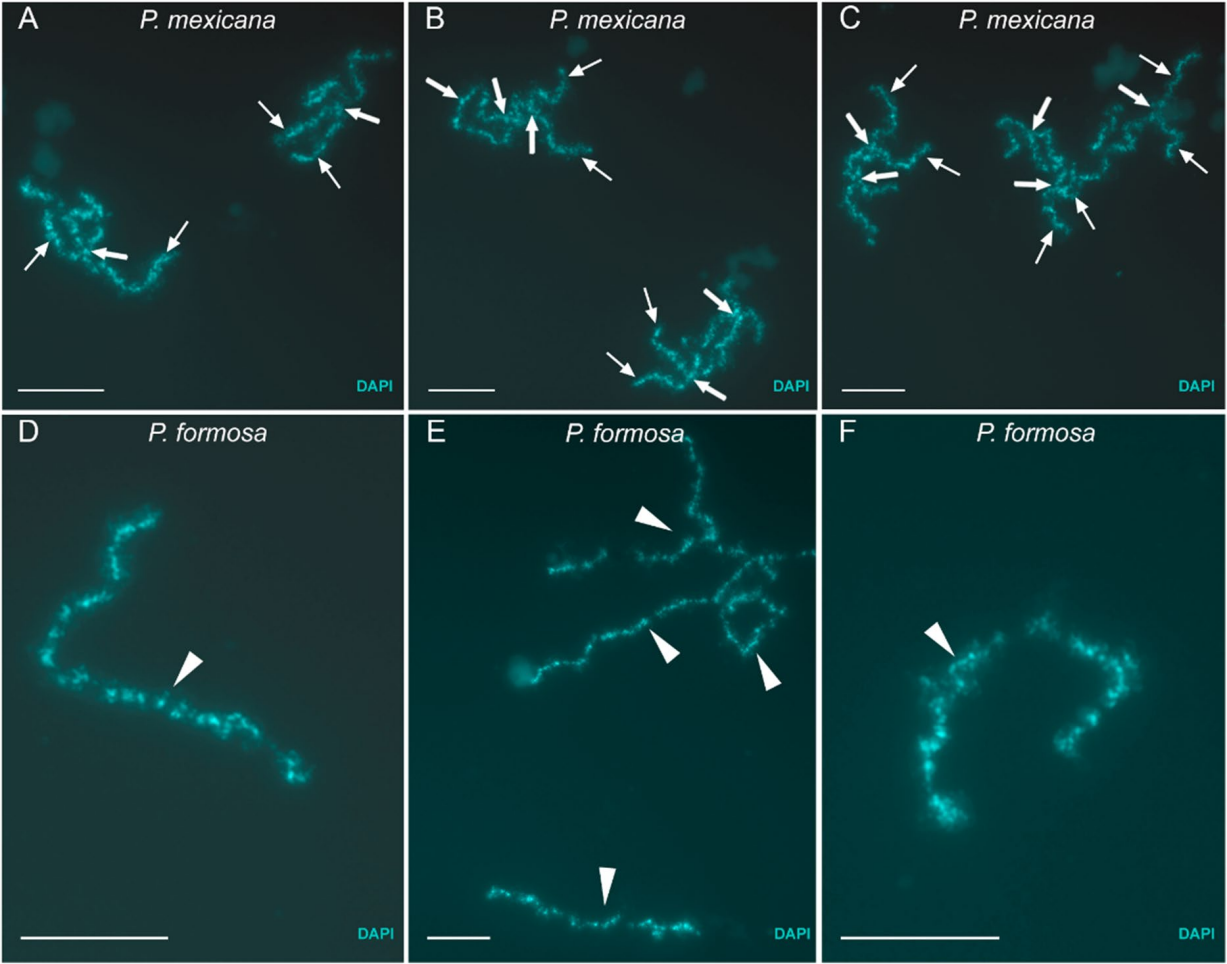
Examples of bivalents (**A**, **B**, **C**) and univalents (**D**, **E**, **F**) from diplotene oocytes of *P. mexicana* (**A**, **B**, **C**) and *P. formosa* (**D**, **E**, **F**). Bivalents (**A**, **B**, **C**; indicated by arrows) selected from full diplotene chromosome spread of *P. mexicana* oocyte presented in Figure S1 A, A`; univalents (**D**, **E**, **F**; indicated by arrowheads) selected from full diplotene chromosome spread of *P. formosa* oocyte presented in Figure S1 B, B’

To validate that regular chromosome pairing is incomplete and chromosomes remain as univalents during *P. formosa* meiotic prophase I, we then studied the oocytes during diplotene stage. In diplotene oocytes spreads from *P. mexicana*, we detected 23 bivalents with homologous chromosomes connected by chiasmata (Fig. 2A–C, Supplementary Fig S1 A, A`, C). In *P. formosa*, we observed only univalents without chiasmata (Fig. 2D–F, Supplementary Fig S1B, B`, D). Analysis of nuclei of intact oocytes revealed the presence of bivalents in oocytes of *P. mexicana* while only univalents were detected in oocytes of *P. formosa* (Supplementary Fig. S2A, B).

### Expression of meiotic genes in sexual and asexual ovaries

**Fig. 3:**
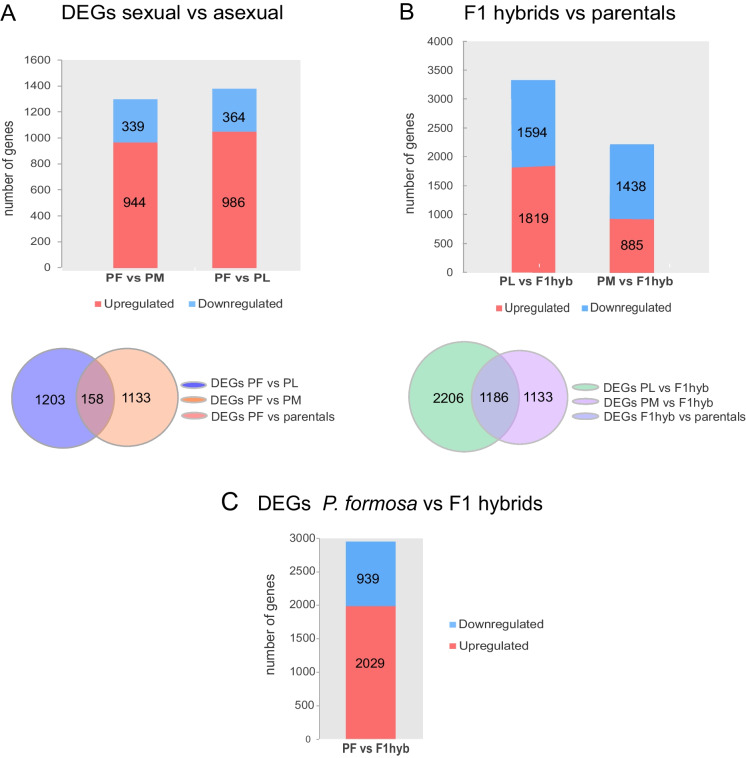
Differentially expressed genes between *P. formosa*, *P. mexicana*, *P. latipinna* and F1 hybrids. Bar plots represent the number of DEGs, and Venn diagrams show the intersection of genes between *P. formosa* and parentals (**A**) and between F1 hybrids and parentals (**B**). Bar plot represents the DEGs between F1 hybrids and *P. formosa* (**C**)

To understand the molecular mechanism why meiosis is initiated but does not proceed to meiotic recombination and formation of bivalents, we first performed comparative transcriptome analyses of ovaries of *P. formosa* and its sexual parental species *P. mexicana* and *P. latipinna* (Fig. 3A, Supplementary Fig. S3). In the sexual parental species, we found that 3327 (2760 protein coding, 557 lncRNA, about 18% of all expressed) genes were differentially expressed (Supplementary Table S1, Fig. S4a). With respect to meiosis genes, *P. mexicana* generally displays higher normalized expression levels. Amongst those higher expressed key meiosis genes were those with a function in the formation of the synaptonemal complex, including *tex 11*, *-12*, *spo11*, *hormad1* and *syce1*, *-2*, *-3* (Supplementary Fig. S4b, Supplementary Table S1).

The comparison of gene expression between *P. formosa* and both parentals showed slightly difference in the number of DEGs (Fig. 3A). Intersecting the transcriptome of *P. formosa* and both parentals revealed 135 differentially expressed protein coding and 23 lncRNA encoding genes (Fig. 3A, Supplementary Table S2-3). The differentially expressed protein coding genes were enriched for GO terms related to immune functions (Supplementary Fig. S4c). For a more in-depth analysis, we generated a manually curated list of 244 genes known to be involved in the stages of female meiosis and oogenesis (Supplementary Table S7-10). We found five downregulated genes in *P. formosa* against *P. mexicana*, four of which are known to be exclusively expressed and thus specific to meiotic prophase I: *spo11*, *tex12*, *meiob* and *syce3*. In addition to this prophase I specific genes, *agt*, a gene involved in the re-entry of metaphase I arrested oocytes in mammals, was also downregulated. Of the eight upregulated genes all are essential for the first meiotic division: *lin28a*, *smc6*, *cbx1*, *cntd1*, *lhcgr*, *tp73*, *fmn2* and *eme1-like* (Supplememtary Table S8). Conversely, we found three meiotic genes upregulated in *P. formosa* in comparison with *P. latipinna*: *hormad1*, *fmn2* and *spyda* (Supplementary Table S9), and no gene was downregulated. In general for the meiotic genes there is an expression bias towards *P. latipinna*.

We found only one gene in common, *formin2*, which was four-fold higher in *P. formosa* when compared with both parentals (Supplementary Table S8-9). This gene is expressed in the developing mammalian oocyte and engages in the homologous chromosome spindle positioning and progression through metaphase of meiosis I (Leader, et al. 2002) but not in the early stage of pairing, synapsis and recombination.

To separate gene expression changes connected to obligate apomixis from DEGs associated with the hybrid situation or automixis, we next analysed meiotic gene expression in F1 hybrids produced from crossing *P. mexicana* females with *P. latipinna* males. The F1 hybrids are not gynogenetic and produce diploid oocytes by terminal fusion. Meiosis I is normal and does not deviate from the sexual parental species (Lampert et al. 2007). Examining the ovary transcriptomes of F1 hybrids vs the parentals revealed 759 differentially expressed protein coding and 427 lncRNA genes (Fig. 3B, Supplementary Table S4-S5). Of these, three have a specific function in meiosis: *cyclinH*, *crossover junction endonuclease EME1-like* and *LIM homeobox8*. Comparing the F1 hybrid to *P. formosa* revealed 2983 protein coding and 154 lncRNA differentially expressed genes (Fig. 3C, Supplementary Table S6). Applying the filter for female meiosis genes, we detected in the hybrids above log2FC |2| nine genes that were downregulated and four up. Of note, *tex11* and *syce3* are also upregulated in the hybrids > log2FC 2 (Supplementary Table S10). The *formin2* gene, which was upregulated in *P. formosa* in relation to the parentals, is also higher compared to the F1 hybrids (Supplementary Table S10).

### Parental allele biased expression in P. formosa ovaries

**Fig. 4:**
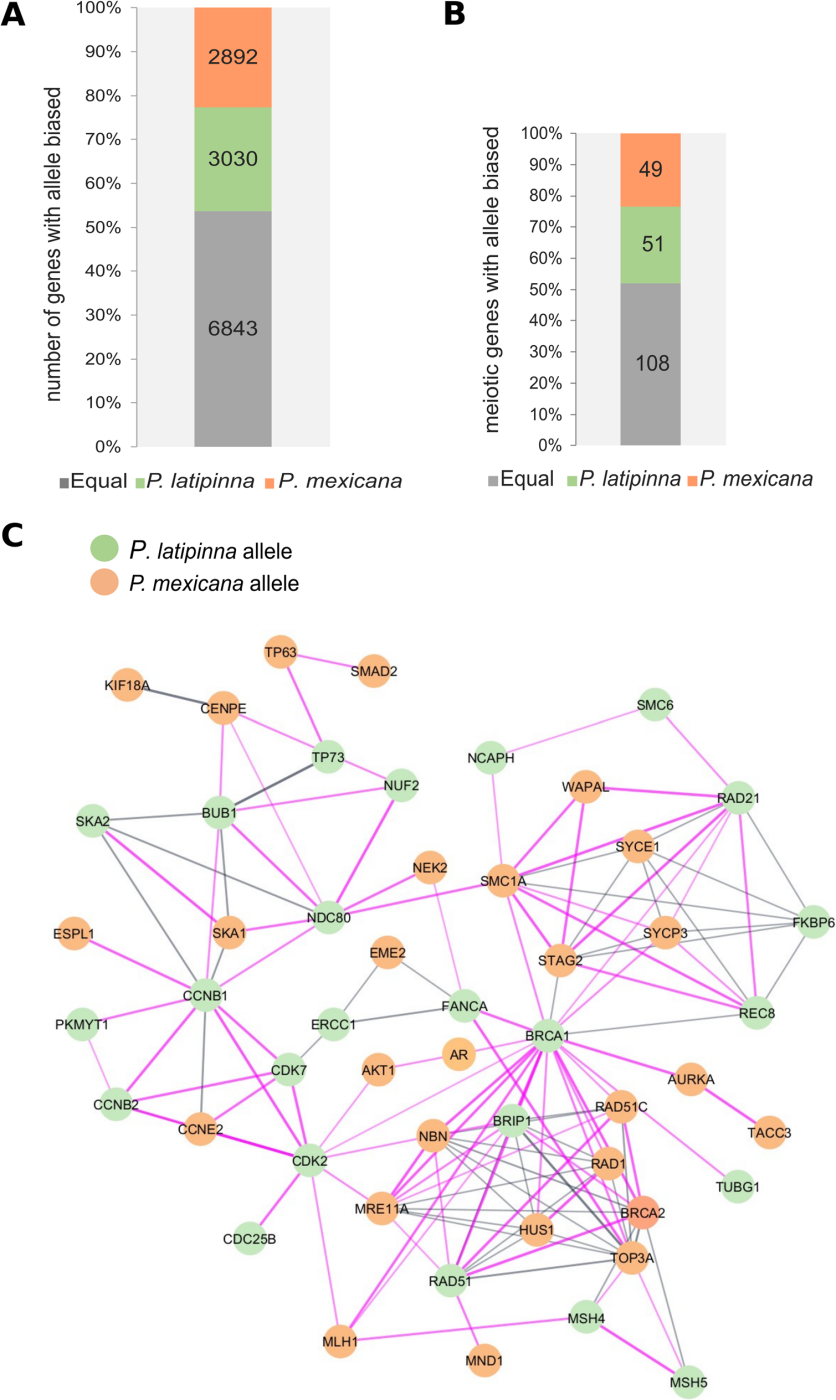
Allele specific gene expression. **A** Stacked bar graphs indicate the percentage of genome wide biased allele expression or equally expressed. **B** Meiotic genes parental allele biased expression categories as a percentage of total number. **C** Protein interaction network of meiotic genes. The nodes represent the specific protein coloured according with the parental biased expression (*P. latipinna* in green, *P. mexicana* in orange). The edges colours represent the type of evidence used to predict protein–protein interactions. Lines in black: co-expression evidence and pink: experimental evidence. The genes on the interaction network are those which present an allele parental biased and for which interactions are known

The Amazon molly is a hybrid between two distantly related species. Hence, allele biased expression or even allele-specific transcription could affect the gene expression profile during meiosis. To approach this question, we assessed unequal expression of the two parental alleles of ovary genes. For 12,765 ovary expressed genes *P. formosa* the parental orthologs could be identified. Allelic expression bias was defined if more than 60% expression came from one parental allele. As result 24% (*n* = 3030) displayed a parental bias for the *P. latipinna* allele, 23% (*n* = 2892) towards the *P. mexicana* allele and 53% were equally expressed from both parental alleles (Fig. 4A).

While the majority of meiosis genes displayed equal distribution of both alleles, 51 genes are biased towards the *P. latipinna* parental genome, and 49 towards the *P. mexicana* parent (Fig. 4B, Supplementary Table S11). Interestingly, at the top of *P. mexicana* biased alleles (> 90%) there is *dmrt1*, an important regulator of male sex determination, but also oogenesis (Zarkower and Murphy 2021). For *P. latipinna* preferentially expressed genes, we found the *mitotic arrest deficient 2-like 2* and the spindle and *kinetochore associated complex subunit 2* genes (Supplementary Table S11).

When the meiotic genes showing allelic expression bias were included in a protein interaction network, many proteins that have an opposite expression bias towards the parental genomes were found as direct interactors, e.g. the synaptonemal complex proteins Sycp3 with the DNA repair protein Brca1 or Rad21 (Fig. 4C). Central to the network, which is characterized by allele biased expression, are proteins that function in DNA repair, recombination and checkpoint of the homologous chromosome segregation.

## Discussion

The cytogenetic analyses by staining of the lateral element protein Sycp3 revealed that in *P. formosa* oocyte chromosomes are forming axial elements of the synaptonemal complex (SC). The absence of Sycp1 proteins could be due to a failure of axial elements assembly or transverse filaments loading. Chromosomes of *P. formosa* may partially align but this does not lead to stable interactions that are transformed to a synaptic configuration during a pachytene stage. Our results corroborate previous observations of SC formation during the early prophase of unisexual *P. formosa* performed by electron microscopy (Monaco et al. 1984). Whether Spo11-driven DSBs are taking place or not is not yet known. However, the absence of chromosome fragmentation and other complex rearrangements together with the absence of Rad51 protein on *P. formosa* pachytene chromosomes suggest that DSBs may not being formed during *P. formosa* meiosis. This implies that recombination repair of programmed DNA breaks that drives chromosome synapsis is most likely not taking place. Mlh1 immunostaining confirms the absence of meiotic crossovers. Consequently, in diplotene stage only univalents with no chiasmata were detected. We were unable to follow up on the chromosome behaviour at the anaphase stage, so we yet cannot confirm from cytogenetics that chromatids rather than homologues are separated. However, the genomic data show that the mature eggs fully retain both parental genomes (Warren et al. 2018). The assembly of the *P. formosa* genome revealed no signs of recombination and both parental haplotypes could be separately assembled at the whole genome level (https://www.ncbi.nlm.nih.gov/assembly/?term=Poecilia+formosa). Together, our present findings indicate that full synapsis is absent and homologous chromosomes do not segregate leading to a diploid egg formed by apomixis.

Gamete formation in apomictic parthenogenesis has been suggested for egg formation in triploid hybrids from *Carassius langsdorfii* and *C. gibelio* species complexes (Kobayasi 1976; Yamashita et al. 1993; YANG et al. 1999). However, in *Carassius* hybrids the first meiotic division is prevented due to the emergence of a tripolar spindle resulting in the failure of first polar body extrusion (Yamashita et al. 1993; YANG et al. 1999). Nevertheless, in contrast to *P. formosa*, in *C. gibelio*, chromosomal pairing and recombination have been observed at least between some homologous chromosomes (Zhang et al. 1992). In other asexuals exploiting endoreplication, only oocytes with normal pairing were able to proceed beyond pachytene while oocytes with aberrant pairing were filtered out at pachytene (Dedukh et al. 2021, 2022). In zebrafish, the mutants with non-functional Mlh1 and Spo11 have different outcomes depending on their sex: males are sterile and produce no sperm while mutant females are fertile, yet produce malformed progeny that fails to develop, likely due to severe aneuploidy (Feitsma et al. 2007; Leal et al. 2008; Blokhina et al. 2019). However, in F1 hybrids of medaka and *Cobitis*, males exhibit aberrant pairing during pachytene, but chromosomes can proceed beyond pachytene until metaphase of meiosis I (Shimizu et al. 1997; Dedukh et al. 2020, 2021). On the contrary, in female hybrids, pachytene cells which exhibit aberrant pairing cannot proceed beyond pachytene to diplotene, thus indirectly indicating the pachytene checkpoint in females (Shimizu et al. 2000; Dedukh et al. 2021). In *P. formosa*, oocytes with univalents are able to proceed beyond pachytene suggesting the absence of a stringent pachytene checkpoint.

The absence of complete synapsis and chromosome reduction of *P. formosa* oocytes is not reflected in the transcriptome data. Comparing the expression of *P. formosa* with the sexual parents revealed only one differentially expressed gene, *formin 2*. This gene participates in the segregation of homologous chromosomes of metaphase I, thus acting after the initial steps of chromosome pairing and recombination. Thus, its overexpression could be a downstream event of the failure of initiating the regular processes of meiosis I. However, we cannot exclude that *formin2* overexpression is due to the somatic cells of the ovary.

Despite meiotic genes are expressed in the asexual *P. formosa*, we noted differences in expression of specific meiosis genes with each of the parentals. Moreover, some of them showed allelic biased expression. One of these, the *spo11* gene, which marks the initiation of recombination at the onset of meiosis (de Massy 2013; Qu et al. 2021) is significantly downregulated compared to the *P. mexicana* parent. This finding is consistent with previous gonadal transcriptome analysis of *P. formosa* (Schedina et al. 2018) in which the number of *spo11* transcripts was lower in comparison to its sexual ancestor *P. mexicana*. The Spo11 exonuclease protein is required for induction of recombination and pairing of meiotic chromosomes (de Massy 2013). From the next stage, chromosome synapsis in meiosis prophase 1, we found that *syce3* and *tex12*, which are major components of the transverse filaments of SC and essential to complete and stabilize full synapsis between homologous chromosomes, are downregulated in *P. formosa* compared with *P. mexicana.* These findings would be in agreement with the notion that although the initiation of axial element assembly of all chromosomes of *P. formosa* appears to be normal, the lack of synapsis appears to be a consequence of the failure to initiate meiotic recombination. It has been shown that the expression level of Spo11 determines the number of DBS and that a sufficient number of DBS are needed to support normal synapsis of chromosomes (Qu et al. 2021).

On the other hand, *sycp1*, *rad51* and *mlh1* are similarly expressed in *P. formosa* as in the parentals; however, there is no protein detected by immunofluorescence. One may have to consider that the regulation of the process that initiates homologous chromosome to synapse occurs at least partially on the protein level. Although we do not see differential expression of the known actors of chromosome alignment and DSB formation, we cannot exclude that so far unknown meiotic genes are misregulated in *P. formosa*.

Notably, genes from the initiation phase of meiosis I are lower expressed in *P. formosa* in comparison with *P. mexicana.* However, meiotic genes follow in general the same expression profile of *P. latipinna*. This is in good agreement with a previous study which noted that a high percentage of the genome shows allele-biased expression towards *P. latipinna* (Lu et al. 2021).

Allelic bias in expression or even allele specific expression could cause either a qualitative difference or, in particular cases of interacting proteins can lead to a disfunction of the protein complex. In a hybrid genome, genes have to interact that underwent lineage-specific diverging evolution in the parental species. If they evolved divergent amino acid sequences, this should interfere with the function of the complex, a phenomenon known as the Bateson-Dobzhansky-Muller (BDM) model of hybrid incompatibility (Orr 1996). Even in cases when high heterozygosity may be generally beneficial for evolution of hybrid species, in order to avoid such incompatibility it may be advantageous to preferentially express one parental allele or even lose the other allele from the genome, especially for genes participating in multimeric protein complexes (Smukowski Heil et al. 2019; Janko et al. 2021). Interestingly, in *P. formosa* approximately 50% of the meiosis genes show allelic expression bias. The interaction network of the differentially expressed meiotic genes may thus be either considerably affected by a hybrid incompatibility effect. Even if single improper interactions may only marginally interfere with protein functions, the multitude of such interactions will amount to and disrupt the regulatory network of meiosis. Alternatively, allele biased expression could indicate a BDM dysgenesis avoidance mechanism to complete oogenesis in a hybrid.

## Conclusions

The cytogenetic analyses indicate that in the Amazon molly the production of unreduced eggs occurs by apomixis due to a failure in the very first steps of meiotic prophase I, leading to univalent formation and no homologous chromosome recombination. The gene expression analysis could not fully explain the behaviour of *P. formosa* univalents during early prophase. Meiotic genes in *P. formosa* are being expressed although synapsis is prevented.

All of the many attempts to replicate the formation of *P. formosa* by crossing *P mexicana* females with *P. latipinna* males have failed so far. Despite female laboratory hybrids from the Amazon molly’s parental species produce diploid eggs, they are not gynogenetic (Lampert et al. 2007). Thus, additional conditions are required. An explanation comes from the rare formation hypothesis for the origin of *P. formosa*, postulating that the right combination of parental alleles that have to come together in the first hybrid is rare (Stöck et al. 2010; Warren et al. 2018). This implicates a polygenic cause for the ability to produce diploid oocytes. Under this hypothesis, many of the identified genes may work together in generating diploid germ cells. Expression changes and incompatibilities in meiosis genes are likely necessary to cause transitions from sexual to parthenogenetic reproduction in hybrid individuals. Therefore, further studies using transcriptomes at a higher resolution such as single-cell RNA-seq and proteomics are required to understand the molecular mechanism of unreduced oocyte formation in *P. formosa.*

## Supplementary Information

Below is the link to the electronic supplementary material.

Supplementary file1 (XLSX 4365 KB)

Supplementary file2 (PDF 755 KB)

## Data Availability

All data referred to are included in the manuscript. The datasets generated during and/or analysed during the current study are available in the supplementary materials and have been submitted to the NCBI BioProject database under accession number PRNJA791764.

## Abbreviations

SC: Synaptonemal complex
DSBs: Double-strand breaks
DEGs: Differentially expressed genes
lncRNA: Long non-coding RNA
FC: Fold change
PCA: Principal component analysis
TPM: Transcript per million
