# Supplementary material for: Achiasmatic meiosis in the unisexual Amazon molly, Poecilia formosa
Source: Chromosome Res. 2022 Dec 2;30(4):443–57. doi: 10.1007/s10577-022-09708-2 (PMC9771850; doi:10.1007/s10577-022-09708-2)
Supplement: Supplementary file 2 — Supplementary file2 (PDF 755 KB) [file 10577_2022_9708_MOESM2_ESM.pdf]

Achiasmatic meiosis in the unisexual Amazon molly, *Poecilia formosa*

Dmitrij Dedukh[1*], Irene da Cruz[2*], Susanne Kneitz[3], Anatolie Marta[1,4], Jenny Ormanns[3], Tomáš Tichopád[1,5], Yuan Lu[6], Manfred Alsheimer[7], Karel Janko[1,8], Manfred Schartl[2,6]

[1] Laboratory of Fish Genetics, Institute of Animal Physiology and Genetics, Czech Academy of Sciences, Liběchov 277 21, Czech Republic

[2]Developmental Biochemistry, Biocenter, University of Wuerzburg, Am Hubland, 97074 Wuerzburg, Germany

[3]Biochemistry and Cell Biology, Biocenter, University of Wuerzburg, Am Hubland, 97074 Wuerzburg, Germany

[4]Institute of Zoology, MD-2028, Academiei 1, 2001 Chisinau, Moldova

[5]University of South Bohemia in České Budějovice, Faculty of Fisheries and Protection of Waters, South Bohemian Research Center of Aquaculture and Biodiversity of Hydrocenoses, Zátiší 728/II, 389 25 Vodňany, Czech Republic

[6]Genetic Stock Center, Texas State University, San Marcos, Texas 78666, USA

[7]Cell and Developmental Biology, Biocenter, University of Wuerzburg, Am Hubland, 97074 Wuerzburg, Germany

[8]Department of Biology and Ecology, Faculty of Science, University of Ostrava, Chittussiho 10, 710 00 Ostrava, Czech Republic

*These authors contributed equally to the work

Correspondence to:

Manfred Schartl

Email: phch1@biozentrum.uni-wuerzburg.de

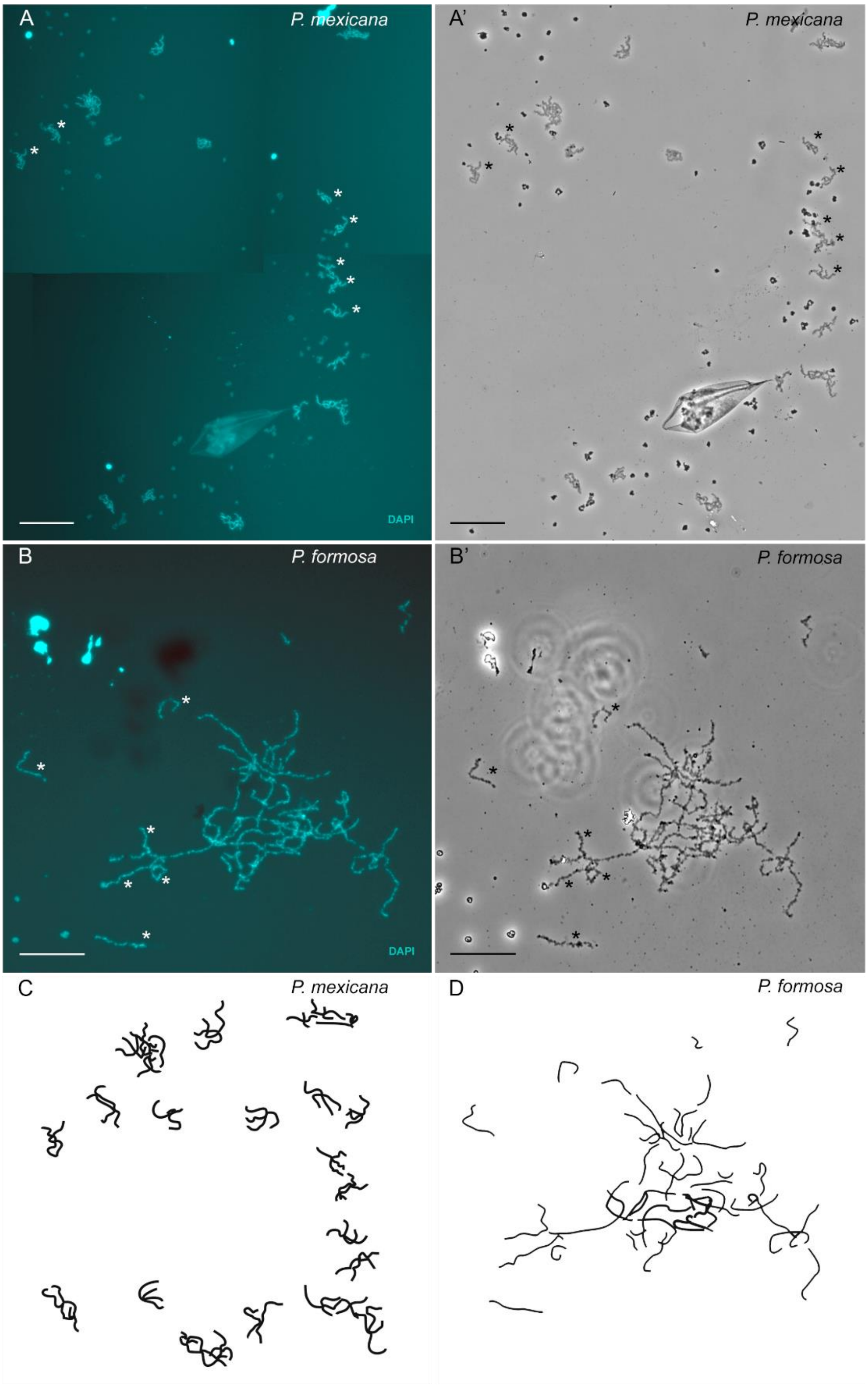

Figure S1. Full diplotene chromosomal spreads from the individual oocytes of *P. mexicana* (**A,**

A') and *P. formosa* (**B**, **B'**). *P. mexicana* diplotene chromosomal spread includes 23 bivalents (**A**, **A'**), *P. formosa* diplotene chromosomal spreads includes approximately 46 univalents. Since the chromosomal spread from individual oocyte was large, four images were taken and merged into one. *P. formosa* diplotene chromosomal spread includes around 46 univalents. Asterisks indicate enlarged bivalents represented in Figure 1 (**G**, **H**, **I)** and **Figure 1** (**J**, **K**, **L**) for *P. mexicana* and *P. formosa* correspondingly. Schematic drawings of full diplotene chromosomal sets with bivalents (**C**) obtained from *P. mexicana* oocyte and with univalents (**D**) obtained from *P. formosa* oocyte. Schematic drawings of full chromosomal sets (**C**) and (**D**) correspond to Figures S1 A, A' and B, B', respectively. Scale bar = 50 µm.

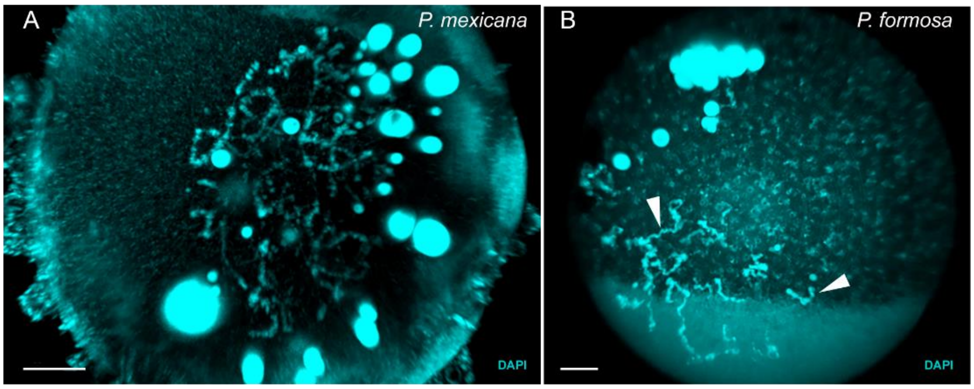

Figure S2. 3D reconstruction of *P. mexicana* (**A**) and *P. formosa* (**B**) diplotene oocytes nuclei after confocal microscopy shows the presence of bivalents (indicated by arrows) and univalents (indicated by arrowheads) correspondingly. Scale bar = 20 µm.

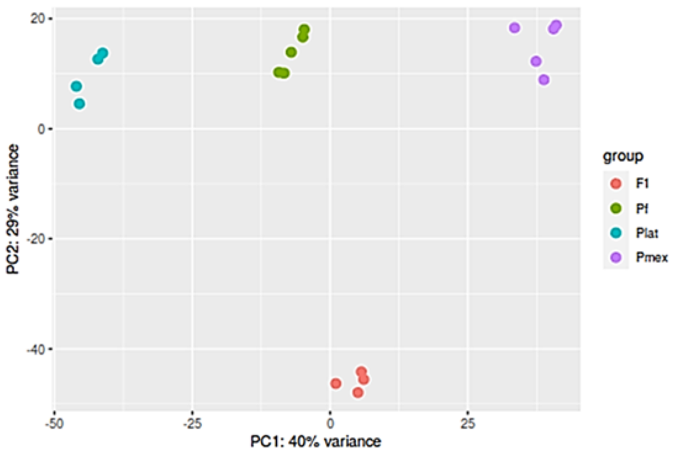

Figure S3. Principal component analysis (PCA) of gene expression on apomictic (*P. formosa*), automictic (F1 hybrids) and sexual parentals (*P. latipinna* and *P. mexicana*). PCA applied to normalized read counts transformed by DESeq. Each dot represents the samples in green for *P. formosa*, blue for *P. latipinna*, violet for *P. mexicana*, and red F1 hybrids. The numbers on the axes represent the respective component (and the % of the variation for each component).

a

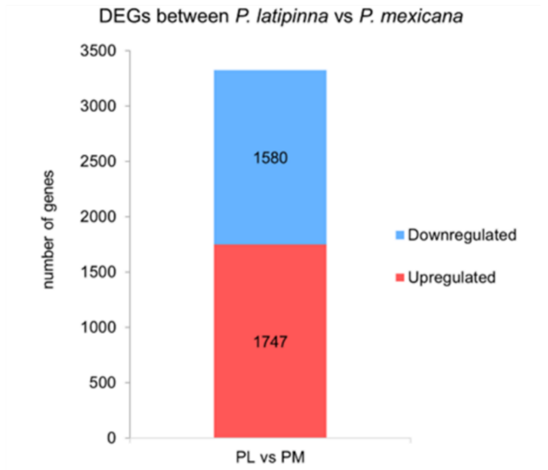

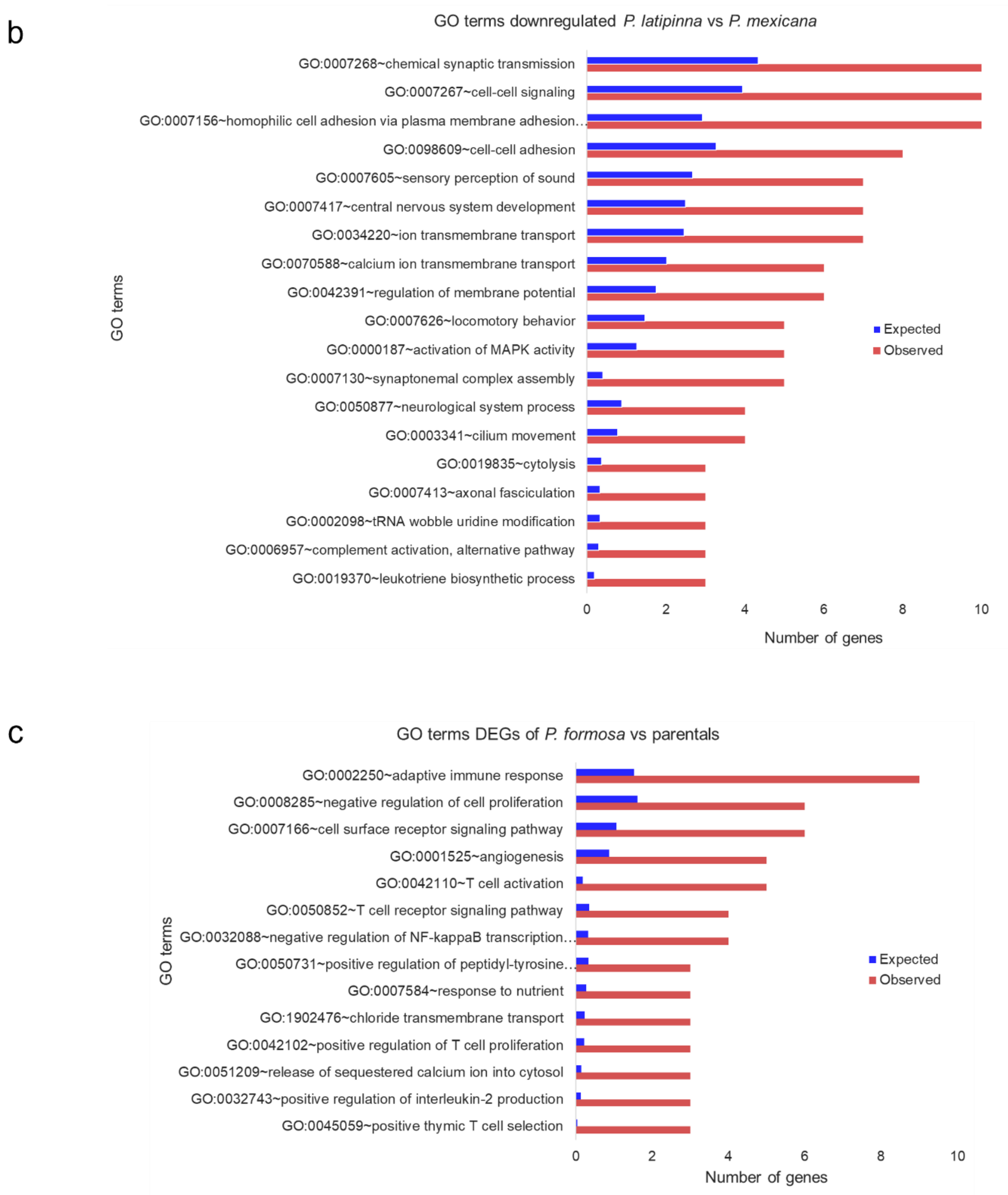

Figure S4. Comparative transcriptome analysis and Gene ontology (GO) enrichment analysis. (a) Differential expressed genes between ovary of *P. latipinna* and *P. mexicana*. (b) GO biological process of *P. latipinna* down-regulated ovary genes when compared with *P. mexicana* (c) GO biological process of differentially expressed genes of ovary among *P. formosa* and parentals.
